# NLRP3 inflammasome activation contributes to VSMC phenotypic transformation and proliferation in hypertension

**DOI:** 10.1038/cddis.2017.470

**Published:** 2017-10-05

**Authors:** Hai-Jian Sun, Xing-Sheng Ren, Xiao-Qing Xiong, Yun-Zhi Chen, Ming-Xia Zhao, Jue-Jin Wang, Ye-Bo Zhou, Ying Han, Qi Chen, Yue-Hua Li, Yu-Ming Kang, Guo-Qing Zhu

**Affiliations:** 1 Department of Physiology, Key Laboratory of Cardiovascular Disease and Molecular Intervention, Nanjing Medical University, Nanjing, Jiangsu 210029, China; 2 Department of Basic Medicine, Wuxi School of Medicine, Jiangnan University, Wuxi, Jiangsu 214122, China; 3 Department of Pathophysiology, Nanjing Medical University, Nanjing, Jiangsu 210029, China; 4 Department of Physiology and Pathophysiology, Cardiovascular Research Center, Xi'an Jiaotong University School of Medicine, Xi'an, Shanxi 710061, China

## Abstract

Inflammation is involved in pathogenesis of hypertension. NLRP3 inflammasome activation is a powerful mediator of inflammatory response via caspase-1 activation. The present study was designed to determine the roles and mechanisms of NLRP3 inflammasome in phenotypic modulation and proliferation of vascular smooth muscle cells (VSMCs) in hypertension. Experiments were conducted in spontaneously hypertensive rats (SHR) and primary aortic VSMCs. NLRP3 inflammasome activation was observed in the media of aorta in SHR and in the VSMCs from SHR. Knockdown of NLRP3 inhibited inflammasome activation, VSMC phenotypic transformation and proliferation in SHR-derived VSMCs. Increased NF*κ*B activation, histone acetylation and histone acetyltransferase expression were observed in SHR-derived VSMCs and in media of aorta in SHR. Chromatin immunoprecipitation analysis revealed the increased histone acetylation, p65-NF*κ*B and Pol II occupancy at the NLRP3 promoter *in vivo* and *in vitro*. Inhibition of NF*κ*B with BAY11-7082 or inhibition of histone acetyltransferase with curcumin prevented the NLRP3 inflammasome activation, VSMC phenotype switching and proliferation in VSMCs from SHR. Moreover, curcumin repressed NF*κ*B activation. Silencing of NLRP3 gene ameliorated hypertension, vascular remodeling, NLRP3 inflammasome activation and phenotype switching in the aorta of SHR. These results indicate that NLRP3 inflammasome activation response to histone acetylation and NF*κ*B activation contributes to VSMC phenotype switching and proliferation and vascular remodeling in hypertension.

Vascular smooth muscle cells (VSMCs) are a dominant cellular constituent of arteries and a critical determinant of vascular disease.^1^ Differentiation and dedifferentiation of VSMCs are essential processes of vascular development.^2^ Unlike skeletal muscle cells and cardiocytes with terminally differentiated feature, VSMCs may preserve phenotype alterations from a differentiated phenotype (contractile phenotype) to a dedifferentiated phenotype (synthetic phenotype) in response to various stimuli.^3^ The phenotypic transformation from differentiated to dedifferentiated VSMCs is involved in reduced expression of contractile proteins, and increased production of extracellular matrix and expression of inflammatory cytokines.^4^ It serves as a major initiating factor for vascular remodeling in several cardiovascular diseases such as atherosclerosis, hypertension, vascular stenosis and diabetic vascular complications.^3^

Chronic vascular inflammation is an important event in the initiation, development and progression of cardiovascular diseases including hypertension, atherosclerosis and abdominal aortic aneurysm.^5, 6, 7^ The low-grade inflammation has been proposed to play a key role in humans and experimental models during the development of hypertension.^8, 9^ Nucleotide-binding oligomerization domain-like receptor protein 3 (NLRP3) inflammasome is a cytosolic complex for early inflammatory responses. It contains NLRP3, apoptosis-associated speck-like protein containing a caspase recruitment domain (ASC) and caspase-1. On activation, NLRP3 forms a complex with its adaptor ASC, which facilitates the conversion of procaspase-1 to active caspase-1. The activated caspase-1 processes pro-interleukin (IL)-1*β* into its mature form IL-1*β* and thus triggers an inflammatory response.^10^ NLRP3 inflammasome is involved in the pathogenesis of a wide variety of diseases, including atherosclerosis, heart failure, metabolic syndrome, diabetic nephropathy, Alzheimer's disease and diabetes.^11, 12, 13^ There is evidence that circulating and vascular levels of pro-inflammatory cytokine IL-1*β* and IL-18 are elevated in hypertension.^14^ However, it is not known whether NLRP3 inflammasome is activated in the VSMCs of spontaneously hypertensive rats (SHR), and whether the inflammasome activation contributes to VSMC phenotypic transformation and proliferation as well as vascular remodeling in hypertension. Moreover, the upstream mechanism of NLRP3 inflammasome activation in SHR is still unknown. The present study was designed to investigate the roles and mechanisms of NLRP3 inflammasome activation in VSMC phenotypic transformation and vascular remodeling in SHR. Furthermore, the effects of NLRP3 gene silencing on hypertension and vascular remodeling were investigated in SHR.

## Results

### NLRP3 inflammasome activation and phenotypic transformation in rat

**Figure 1:**
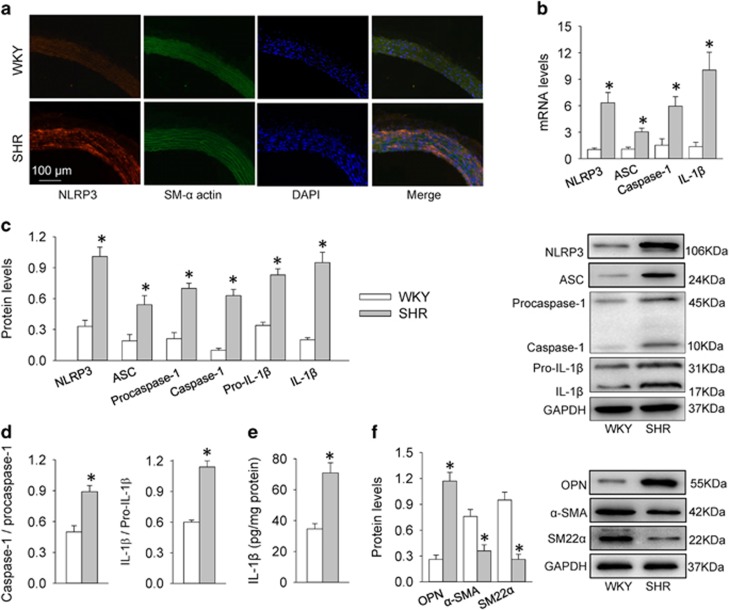
NLRP3 inflammasome activation and phenotypic transformation in the aortic media of WKY and SHR. (**a**) Immunofluorescence double staining showing the overlap of NLRP3 (red) and SM-*α* actin (green) in aorta. Nuclei were stained by DAPI (blue). (**b**) Relative mRNA levels of NLRP3, ASC, caspase-1 and IL-1*β* in media of aorta. (**c**) Relative protein expressions of NLRP3, ASC, procaspase-1, caspase-1, pro-IL-1*β* and IL-1*β* in media of aorta. (**d**) Ratio of caspase-1 to procaspase-1 and ratio of IL-1*β* to pro-IL-1*β*. (**e**) IL-1*β* levels measured with enzyme-linked immunosorbent assay. (**f**) Expressions of synthetic protein (OPN) and contractile proteins (*α*-SMA, SM22*α*) in media of aorta. Values are mean±S.E. *P<0.05 *versus* WKY. *n*=6

Immunofluorescence double staining showed that NLRP3 immunoreactivity in aortic media was increased in SHR compared with WKY (Figure 1a). The mRNA levels of NLRP3, ASC, caspase-1 and IL-1*β* in aortic media were increased in SHR compared with those in WKY (Figure 1b). The protein levels of NLRP3, ASC, procaspase-1, caspase-1, pro- IL-1*β* and mature IL-1*β* in aortic media were upregulated in SHR (Figure 1c). The NLRP3 inflammasome activation was further confirmed by the increased ratio of caspase-1 to procaspase-1 and the ratio of IL-1*β* to pro-IL-1*β* (Figure 1d) as well as the increased IL-1*β* levels in aortic media in SHR (Figure 1e). VSMC phenotypic transformation is characterized by an increase in synthetic protein including OPN and a reduction in contractile proteins such as *α*-smooth muscle actin (*α*-SMA) and smooth muscle 22*α* (SM22*α*) in hypertension.^15, 16^ Contractile proteins *α*-SMA and SM22*α* were downregulated, while synthetic protein osteopontin (OPN) were upregulated, indicating phenotypic transformation in aortic media of SHR (Figure 1f).

### Effects of NLRP3 knockdown in VSMCs

**Figure 2:**
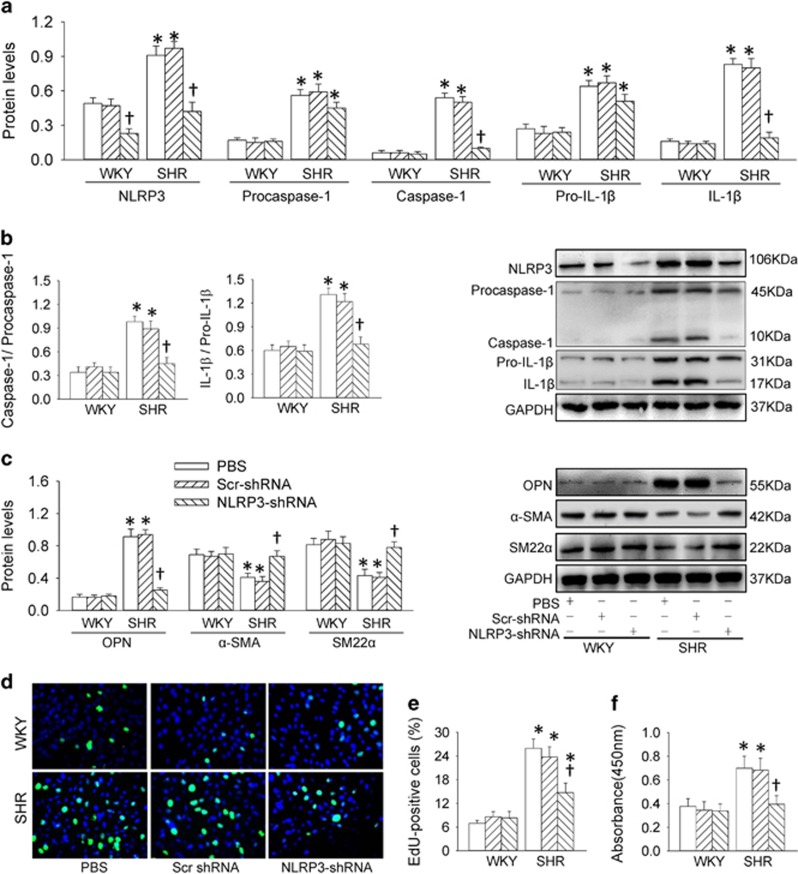
Effects of NLRP3 knockdown on NLRP3 inflammasome activation, phenotypic transformation and proliferation in VSMCs from aortas of WKY and SHR. NLRP3 knockdown was conducted with shRNA (1 × 10^7^ infectious units for 48 h). (**a**) Relative protein expressions of NLRP3, procaspase-1, caspase-1, pro-IL-1*β* and IL-1*β*. (**b**) Ratio of caspase-1 to procaspase-1 and ratio of IL-1*β* to pro-IL-1*β*. (**c**) Relative protein expressions of OPN, *α*-SMA and SM22*α*. (**d**) Representative images showing EdU-positive cells measured with Edu incorporation assay. Blue fluorescence shows cell nuclei and green fluorescence stands for cells with DNA synthesis. (**e**) Bar graph showing the percentage of EdU-positive cells. (**f**) VSMC proliferation was evaluated with changes of absorbance measured with CCK-8 kits. Values are mean±S.E. *P<0.05 *versus* WKY; ^†^*P*<0.05 *versus* PBS or Scrambled (Scr)-shRNA. *n*=6

The efficiency of NLRP3 knockdown with shRNA was confirmed by reduced NLRP3 expression in VSMCs of SHR (Supplementary Figure S1). NLRP3 knockdown attenuated the upregulation of NLRP3, caspase-1 and IL-1*β* protein expressions, but had no significant effects on procaspase-1 and pro-IL-1*β* in VSMCs from SHR (Figure 2a). Caspase-1 activity was increased in SHR, which was prevented by NLRP3 knockdown (Supplementary Figure S2A). NLRP3 knockdown prevented the increases in the ratio of caspase-1 to procaspase-1 and the ratio of IL-1*β* to pro-IL-1*β* (Figure 2b). VSMC phenotypic transformation in SHR was rescued by the NLRP3 partial deletion with shRNA (Figure 2c). NLRP3 knockdown prevented the increased proliferative capacity in VSMCs from SHR, evidenced by the reduced number of 5-ethynyl-2′-deoxyuridine (EdU)-positive cells (Figures 2d and e), absorbance (Figure 2f) and PCNA expression (Supplementary Figure S2B). On the other hand, Ang II plays an important roles in vascular inflammation.^17^ Blockage of AT_1_ receptors with losartan attenuated but could not abolished the NLRP3 inflammasome activation in VSMCs from aortas of SHR (Supplementary Figures S3A and B), suggesting that activation of AT_1_ receptors only partially contributed the NLRP3 inflammasome activation in SHR.

### Analysis of promoter region of NLRP3 in VSMCs

**Figure 3:**
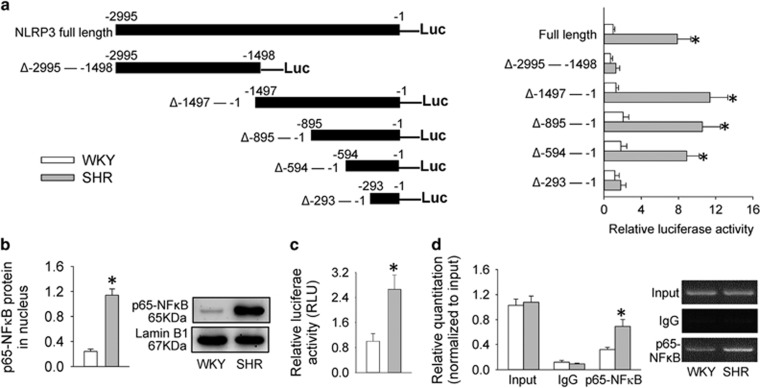
Identification of the region of NLRP3 promoter for NLRP3 induction and evaluation of the expression and activity of NF*κ*B in hypertension. (**a**) Relative luciferase activity derived from series of deletion mutants of NLRP3 promoter constructs in VSMCs. (**b**) Relative protein expressions of p65-NF*κ*B expression in nucleus of VSMCs. (**c**) Relative luciferase activity after VSMCs were transfected with NF*κ*B luciferase reporter gene for 48 h. (**d**) Relative quantitation of precipitated DNA determined with chromatin immunoprecipitation analysis. Values are mean±S.E. *P<0.05 *versus* WKY. *n*=6

Luciferase activity derived from series of deletion mutants of NLRP3 promoter constructs was examined to determine the primary promoter region of NLRP3 in VSMCs. The luciferase activity in full-length promoter region of NLRP3 gene was higher in VSMCs from SHR than those from WKY. The NLRP3 transcription was activated only when a small region (−594 to −294) is preserved in SHR-derived VSMCs (Figure 3a). According to the Promoter Scan from the Bioinformatics and Molecular Analysis Section (BIMAS) of NIH, a promoter finding and analysis program on the internet (http://www-bimas.cit.nih.gov/molbio/proscan/), the putative NF*κ*B-binding sites may be present within the region from −594 to −294 bp in the NLRP3 promoter.

### NF*κ*B signaling in VSMCs

**Figure 4:**
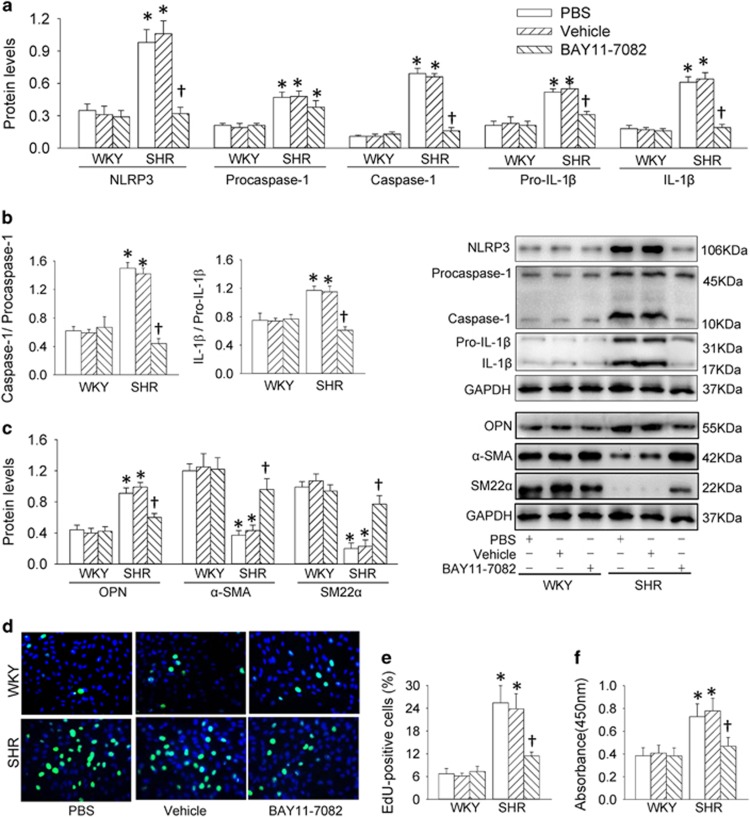
Effects of a NF*κ*B inhibitor BAY11-7082 (10 *μ*M for 48 h) on NLRP3 inflammasome activation, phenotypic transformation and proliferation in VSMCs from aortas of WKY and SHR. (**a**) Relative protein expressions of NLRP3, procaspase-1, caspase-1, pro-IL-1*β* and IL-1*β*. (**b**) Ratio of caspase-1 to procaspase-1 and ratio of IL-1*β* to pro-IL-1*β*. (**c**) Relative protein expressions of OPN, *α*-SMA and SM22*α*. (**d**) Representative images showing EdU-positive cells measured with Edu incorporation assay. Blue fluorescence shows cell nuclei and green fluorescence stands for cells with DNA synthesis. (**e**) Bar graph showing the percentage of EdU-positive cells. (**f**) VSMC proliferation was evaluated with changes of absorbance measured with CCK-8 kits. Values are mean±S.E. *P<0.05 *versus* WKY; ^†^*P*<0.05 *versus* PBS or Vehicle. *n*=6

The levels of p65-NF*κ*B in nucleus (Figure 3b) and the activity of NF*κ*B luciferase reporter gene (Figure 3c) were increased in SHR-derived VSMCs. Chromatin immunoprecipitation (ChIP) analysis showed that the bindings of p65-NF*κ*B to the NLRP3 promoter were increased in SHR-derived VSMCs (Figure 3d). These results suggest that NF*κ*B signaling in VSMCs is activated in SHR. Thus, an NF*κ*B inhibitor BAY11-7082 was used to determine whether NF*κ*B signaling in VSMCs would contribute to NLRP3 inflammasome activation and phenotypic transformation in hypertension. BAY11-7082 almost normalized the increased NLRP3, caspase-1, pro-IL-1*β* and IL-1*β* expressions (Figure 4a) and caspase-1 activity (Supplementary Figure S4A), but no significant effects on procaspase-1 expression in SHR-derived VSMCs. It prevented the increased ratio of caspase-1 to procaspase-1 and IL-1*β* to pro-IL-1*β* (Figure 4b), as well as the phenotypic transformation in VSMCs of SHR (Figure 4c). Inhibiting NF*κ*B attenuated VSMC proliferation in VSMCs from SHR, indicated by the reduced number of EdU-positive cells (Figures 4d and e), absorbance (Figure 4f) and PCNA expression (Supplementary Figure S4B).

### Histone acetylation in VSMCs

**Figure 5:**
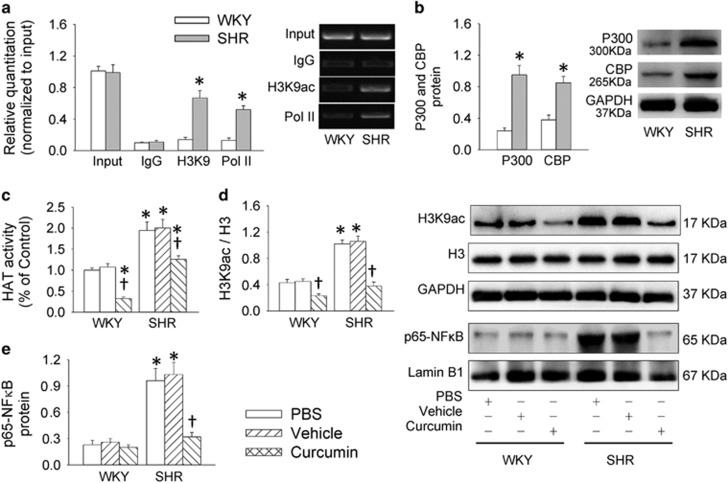
Roles of histone acetylation in NF*κ*B and NLRP3 activation in VSMCs from aortas of WKY and SHR. (**a**) Enrichment of acetylated histone H3K9 and Pol II in the NLRP3 promoter. (**b**) Expressions of histone acetyltransferase (HAT) CBP and P300. (**c**) Effects of an HAT inhibitor curcumin (20 *μ*M for 48 h) on HAT activity. (**d**) Effects of an HAT inhibitor curcumin on histone acetylation. (**e**) Effects of curcumin on p65-NF*κ*B in nucleus. Values are mean±S.E. *P<0.05 *versus* WKY; ^†^*P*<0.05 *versus* PBS or Vehicle. *n*=4

**Figure 6:**
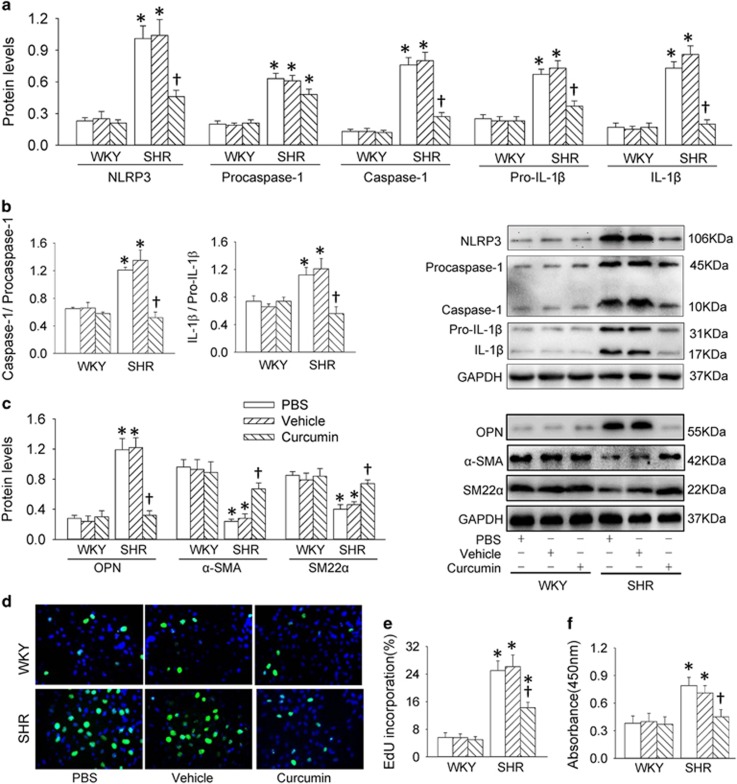
Effects of a histone acetyltransferase inhibitor curcumin (20 *μ*M for 48 h) on NLRP3 inflammasome activation, phenotypic transformation and proliferation in VSMCs from aortas of WKY and SHR. (**a**) Relative protein expressions of NLRP3, procaspase-1, caspase-1, pro-IL-1*β* and IL-1*β*. (**b**) Ratio of caspase-1 to procaspase-1 and ratio of IL-1*β* to pro-IL-1*β*. (**c**) Relative protein expressions of OPN, *α*-SMA and SM22*α* and PCNA. (**d**) Representative images showing EdU-positive cells measured with Edu incorporation assay. Blue fluorescence shows cell nuclei and green fluorescence stands for cells with DNA synthesis. (**e**) Bar graph showing the percentage of EdU-positive cells. (**f**) VSMC proliferation was evaluated with changes of absorbance measured with CCK-8 kits. Values are mean±S.E. *P<0.05 *versus* WKY; ^†^*P*<0.05 *versus* PBS or Vehicle. *n*=6

Histone acetylation is identified as a stimulator for NF*κ*B activation.^18^ ChIP analysis revealed that acetyl histone H3 modification and Pol II occupancy at the NLRP3 promoter were increased in VSMCs from SHR (Figure 5a). VSMCs from SHR showed an upregulated histone acetyltransferase (HAT) including EP300-binding protein (p300) and CREB-binding protein (CBP) in SHR-derived VSMCs (Figure 5b). Curcumin, an inhibitor of histone acetyltransferases, suppressed the increased HAT activity (Figure 5c), histone modifications of acetylation in histone H3 (Figure 5d) and NF*κ*B activation (Figure 5e) in SHR-derived VSMCs. Curcumin suppressed the upregulation of NLRP3, caspase-1, pro-IL-1*β* and IL-1*β* proteins (Figure 6a), the increased ratio of caspase-1 to procaspase-1 and IL-1*β* to pro-IL-1*β* (Figure 6b), as well as the enhanced caspase-1 activity (Supplementary Figure S5A), but had no significant effect on procaspase-1 expression (Figure 6a) in SHR-derived VSMCs. Moreover, curcumin attenuated the VSMC phenotypic transformation (Figure 6c), and prevented proliferation, evidenced by the reduced number of EdU-positive cells (Figures 6d and e), absorbance (Figure 6f) and PCNA expression (Supplementary Figure S5B) in VSMCs from SHR.

### Histone acetylation, NF*κ*B-p65 expression and NLRP3 promoter complexes in rats

In light of the above-mentioned studies *in vitro*, we conclude that histone acetylation contributes to NLRP3 inflammasome activation via NF*κ*B in VSMCs of SHR. Thus, the histone acetylation and NF*κ*B activation in aortic media of WKY and SHR were further examined. Similarly, the acetylation at lysine 9 of histone 3 (H3K9ac), the CBP and P300 expression of histone acetyltransferase and the p65-NF*κ*B expression in nucleus were increased in the aortic media of SHR compared with that of WKY (Supplementary Figure S6). ChIP analysis confirmed the enrichment of acetyl histone H3 modification p65 and Pol II within the NLRP3 promoter in the aortic media of SHR (Supplementary Figure S7).

### Effects of HAT inhibition on vascular remodeling in SHR

**Figure 7:**
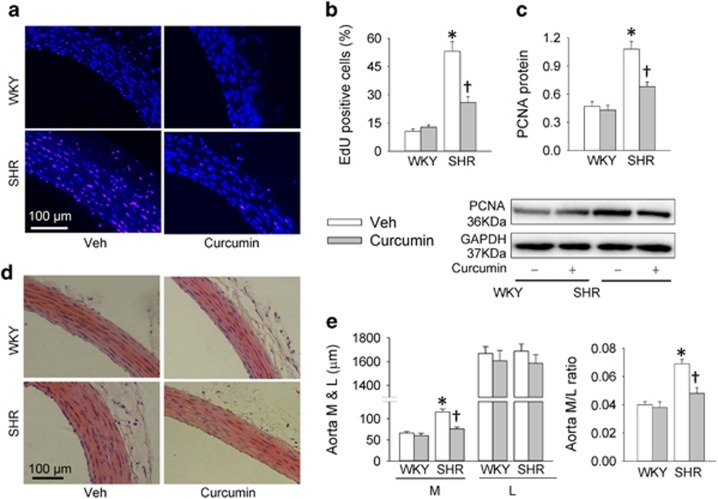
Effects of a histone acetyltransferase inhibitor curcumin on vascular remodeling in SHR. The measurements were made 2 weeks after transfection. WKY and SHR were subjected to intragastric administration of polyethylene glycol (Veh) or curcumin (100 mg/kg/day) for 2 weeks. (**a**) Representative images showing EdU-positive cells measured with Edu incorporation assay. Blue fluorescence shows cell nuclei and red fluorescence stands for cells with DNA synthesis. (**b**) Bar graph showing the percentage of EdU-positive cells. (**c**) Relative protein expressions of PCNA. (**d**) Representative sections of thoracic aortas with hematoxylin–eosin staining. (**e**) Media thickness (**m**), lumen diameter (**l**) and the ratio of M to L of aorta. Values are mean±S.E. *P<0.05 *versus* WKY; ^†^*P*<0.05 *versus* Veh. *n*=6

Intragastric administration of curcumin for 2 weeks was used to evaluate the effects of HAT inhibition on vascular remodeling in SHR. Curcumin had no significant effect on the number of EdU-positive cells and the PCNA protein expression in aortic media of WKY, but reduced the number of EdU-positive cells (Figures 7a and b) and the PCNA protein expression (Figure 7c) in aortic media of WKY. Furthermore, curcumin reduced the media thickness and the ratio of media thickness to lumen diameter in aorta of SHR (Figures 7d and e).

### Effects of NLRP3 gene silencing on vascular remodeling in SHR

**Figure 8:**
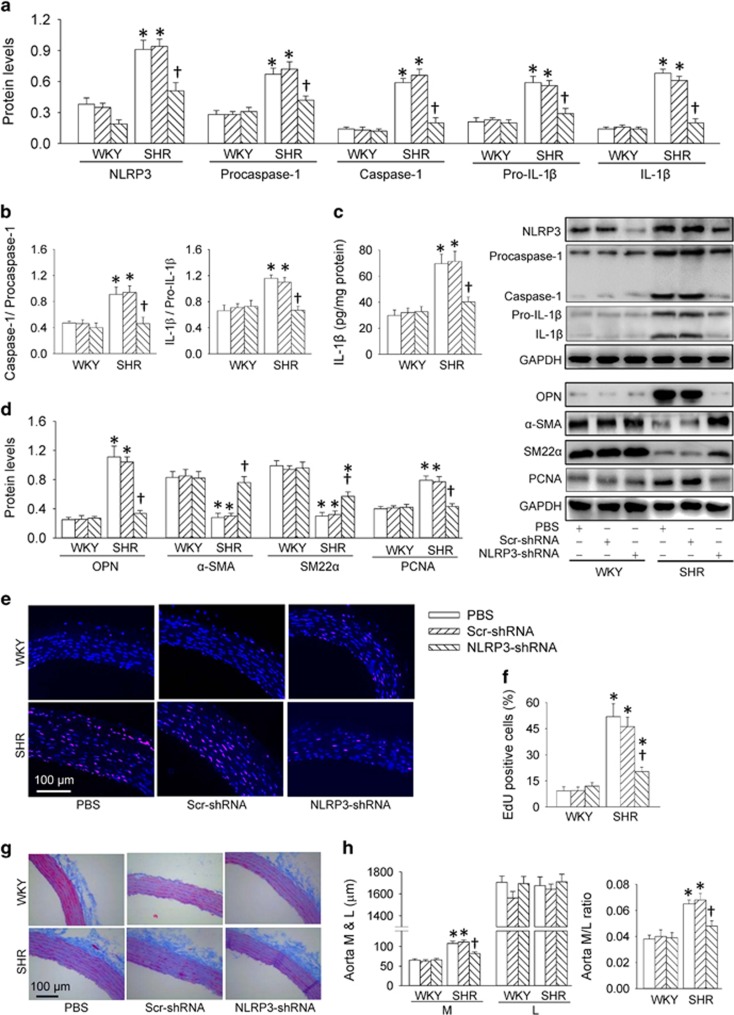
Therapeutical effect of NLRP3 knockdown on NLRP3 inflammasome activation, phenotypic transformation, vascular remodeling and hypertension in SHR. The measurements were made 2 weeks after transfection. (**a**) Relative protein expressions of NLRP3, procaspase-1, caspase-1, pro-IL-1*β* and IL-1*β*. (**b**) Ratio of caspase-1 to procaspase-1 and ratio of IL-1*β* to pro-IL-1*β*. (**c**) IL-1*β* levels measured with enzyme-linked immunosorbent assay. (**d**) Relative protein expressions of OPN, *α*-SMA, SM22 *α* and PCNA. (**e**) Representative images showing EdU-positive cells measured with EdU incorporation assay. Blue fluorescence shows cell nuclei and red fluorescence stands for cells with DNA synthesis. (**f**) Bar graph showing the percentage of EdU-positive cells. (**g**) Representative sections of thoracic aortas with Masson staining. (**h**) Media thickness (M), lumen diameter (L) and the ratio of M to L of aorta. Values are mean±S.E. *P<0.05 *versus* WKY; ^†^*P*<0.05 *versus* PBS or scrambled (Scr-) shRNA. *n*=6

Adenovirus harboring shRNA against NLRP3 was intravenously administered to assess the therapeutical effects of NLRP3 knockdown on vascular remodeling in SHR. NLRP3 protein in aortic media was upregulated in SHR, which was reduced by the NLRP3-shRNA introduction, peaking at 2 weeks after intervention (Supplementary Figure S8A). NLRP3-shRNA reduced blood pressure in SHR, but not in WKY. However, it had no significant effect on heart rate (Supplementary Figure S8B). NLRP3-shRNA not only downregulated the NLRP3 protein, but also the procaspase-1, caspase-1, pro-IL-1*β* and IL-1*β* protein expressions in SHR (Figure 8a). Moreover, knockdown of NLRP3 reduced the ratio of caspase-1 to procaspase-1 and IL-1*β* to pro-IL-1*β* (Figure 8b), as well as the IL-1*β* levels (Figure 8c). The upregulated synthetic protein OPN and the downregulated contractile proteins *α*-SMA and SM22*α* in SHR were reduced by NLRP3-shRNA intervention, suggesting that NLRP3 knockdown attenuates VSMC phenotypic transformation (Figure 8d). On the other hand, the proliferation of vascular smooth in SHR was inhibited by NLRP3 knockdown, evidenced by the reduced PCNA expression (Figure 8d) and the reduced EdU-positive cells (Figures 8e and f). Importantly, NLRP3 gene silencing reduced the media thickness and the ratio of media thickness to lumen diameter in the aorta of SHR (Figures 8g and h).

## Discussion

Vascular inflammation is considered to play a critical role in vascular remodeling in several vascular diseases such as hypertension and atherosclerosis.^5, 8, 9^ Plasma IL-1*β* level was increased in stroke-prone SHR^19^ and renovascular hypertensive rats.^20^ IL-1*β* accelerated the onset of stroke concomitant with severe hypertension,^19^ and stimulated the VSMC proliferation.^21^ The present study provides new insights that NLRP3 inflammasome activation contributes to the VSMC phenotypic transformation, proliferation and vascular remodeling in SHR. Excessive histone H3 acetylation facilitates NF*κ*B transactivation, and increased NF*κ*B and Pol II binding to the NLRP3-promoter region, and then stimulates NLRP3 inflammasome activation. Inhibition of histone acetyltransferases or knockdown of NLRP3 attenuates NLRP3 inflammasome activation and vascular remodeling in SHR.

Deregulation of VSMC phenotypic transformation is responsible for the development and progression of hypertension and its related vascular pathologies.^22^ NLRP3 inflammasome is important for caspase-1 activation and IL-1*β* release.^10, 23, 24^ In the present study, NLRP3 activation, inflammation and phenotypic transformation were found in the SHR, which were attenuated by NLRP3 knockdown in SHR-derived VSMCs, or by NLRP3 gene silencing in the aortic media of SHR. Both *in vivo* and *in vitro* studies showed that the NLRP3 is critical in the development of vascular inflammation and VSMC phenotypic transformation in hypertension. NLRP3 may be a critical target for attenuation of chronic vascular inflammation in hypertension.

NLRP3 inflammasome can be activated by a wide range of danger signals that derive not only from microorganisms but also from a variety of signals and metabolic dysregulation such as Ca^2+^ signaling, reactive oxygen species (ROS), nitric oxide (NO), Ang II, endoplasmic reticulum stress and mitochondrial dysfunction.^25, 26^ However, the mechanisms of NLRP3 inflammasome activation in hypertension are not well known. Ang II is important in inducing vascular inflammation.^17^ NLRP3 inflammasome activation is involved in Ang II-induced kidney damage.^27^ We found that AT_1_ receptor activation in the VSMCs only played a partially role in the NLRP3 inflammasome activation in the SHR. NF*κ*B is known to a necessary prerequisite for NLRP3 inflammasome activation in primary hepatocytes.^28^ Inhibition of NF*κ*B reduced the expression of NLRP3 inflammasome in peripheral blood mononuclear cells.^29^ In the present study, NF*κ*B signal was activated, and the region from −594 to −294 bp in the NLRP3 promoter was mainly responsible for NLRP3 expression in SHR-derived VSMCs. Inhibition of NF*κ*B prevented the NLRP3 inflammasome activation, phenotypic transformation and proliferation in VSMCs from SHR. These findings revealed that the sustained transcriptional activity of NLRP3 was dependent on the enhanced binding of transcriptional factors NF*κ*B to the NLRP3 promoter in hypertension. NF*κ*B activation is critical for NLRP3 inflammasome activation, phenotypic transformation and proliferation in VSMCs from SHR.

Epigenetic modifications have been considered as key contributors to control targeted gene expression in both physiological and pathophysiological conditions. Histone acetylation via histone acetyltransferase CBP/p300 contributes to active transcription via rendering gene promoters more accessible to the transcription machinery. Acetylation of histone H3 and p300 was involved in the platelet-derived growth factor-BB-mediated VSMC proliferation.^30^ Post-translational modifications such as acetylation of histone H3 augmented p65 activity.^31^ We found that the bindings of histone acetylation, p65 and Pol II to the NLRP3 promoter were increased in both aortic media in SHR and SHR-derived VSMCs. The HAT protein expression and activity and the acetylation of histone H3 were increased in SHR-derived VSMCs. Inhibition of HAT with curcumin prevented the NF*κ*B activation and subsequent NLRP3 inflammasome activation, VSMC phenotypic transformation and proliferation in the VSMCs from SHR. The results indicate that the HAT activation and the following NF*κ*B and NLRP3 inflammasome activation are important contributors in the VSMC phenotypic transformation and proliferation in hypertension. The findings were further supported by the evidence that persistent intragastric administration of curcumin to inhibit HAT attenuated the proliferation of vascular smooth muscle and vascular remodeling in SHR.

Vascular remodeling in hypertension may initially be adaptive, but eventually it becomes maladaptive and contributes to the development and complications of hypertension.^32, 33^ VSMC phenotypic transformation is as a major initiating factor for vascular remodeling in hypertension.^3^ VSMC proliferation are closely linked with vascular remodeling and hypertension.^34^ Therefore, the therapeutical effects of NLRP3 gene silencing on vascular remodeling and hypertension were examined in SHR. We found that silencing of NLRP3 gene caused a moderate depressor effect in SHR. It inhibited NLRP3 inflammasome activation and inflammation, VSMC phenotypic transformation and proliferation, as well as vascular remodeling in the aortas of SHR. These results indicate that NLRP3 inflammasome activation plays an important role in the hypertension and vascular remodeling. NLRP3 may be a novel target for the intervention of hypertension and vascular remodeling. A limitation in the present study is that we cannot determine whether the antihypertensive effect of NLRP3 gene silencing is secondary to the improvement of vascular remodeling.

In conclusion, NLRP3 inflammasome is a critical positive regulator of VSMC phenotypic transformation and proliferation in hypertension. Increased histone acetylation and subsequent NF*κ*B activation in hypertension contribute to the NLRP3 inflammasome formation and activation. NLRP3 knockdown reduces blood pressure, inhibits VSMC inflammation, phenotypic transformation and proliferation, and attenuates vascular remodeling in SHR. NLRP3 inflammasome plays an important role in hypertension and vascular remodeling. NLRP3 may be a novel target for the intervention of hypertension and vascular remodeling.

## Materials and methods

Male WKY and SHR aged 12 weeks (Vital River Laboratory Animali Technology Co. Ltd, Beijing, China) were used in the present study. Experiments were approved by the Experimental Animal Care and Use Committee of Nanjing Medical University. The procedures were conformed to the Guide for the Care and Use of Laboratory Animal published by the US National Institutes of Health (NIH publication, 8th edition, 2011). Animals were housed in a temperature-controlled room with a 12-h light/dark cycle and a free access to standard chow and tap water.

### VSMC culture

Primary VSMCs were isolated from thoracic aorta of WKY and SHR aged at 8 weeks. VSMCs were cultured in Dulbecco’s modified Eagle’s medium (DMEM) with 10% fetal bovine serum (FBS, Hyclone, Logan, UT, USA), penicillin (100 IU/ml) and streptomycin (100 mg/ml) at 37 °C in a 5% CO_2_ humidified incubator. Cells in the second to sixth passages were used, and cells at 80–90% confluence were arrested by incubating in serum-deprived DMEM for 24 h before intervention.^35^

### Immunohistochemistry

The aorta was fixed in 4% formaldehyde, embedded in paraffin and transversely cut into 5-*μ*m sections using a cryostat (Leica, Solms, Germany). The sections were washed three times with 0.1 M PBS after deparaffinization, and blocked with blocking buffer (Dual Endogenous Enzyme Block; Dako, Carpinteria, CA, USA) for 5 min. The sections were incubated with goat primary anti-NLRP3 antibody (1:100; Abcam, Cambridge, UK) for 24 h at 4 °C, followed by incubation with horseradish peroxidase-conjugated rabbit anti-goat IgG for 30 min in room temperature. 3,3-Diaminobenzidine was used to develop the positive cells in arteries. Sections were counterstained with hematoxylin, and then covered with glass coverslips with xylene-based mounting medium.

### Dual immunofluorescence

Paraffin-embedded sections were permeabilized with 0.1% Triton X-100 in PBS after deparaffinization and rehydration, following by washing with PBS three times. The sections were incubated with goat anti-NLRP3 antibody (1:100) or rabbit anti-SM *α*-actin (1:50; Sigma-Aldrich, St. Louis, MO, USA), and then secondary TRITC-conjugated goat anti-rabbit IgG (1:400) or FITC-conjugated monkey anti-goat IgG (1:200) (Life Technologies, Gaithersburg, MD, USA), respectively. For nuclear staining, DAPI with mounting medium (Vector Laboratories, Inc., Burlingame, CA, USA) was used after immunofluorescence staining. The fluorescence signals were captured by fluorescence microscopy (DX51; Olympus, Tokyo, Japan).

### Masson’s staining and hematoxylin–eosin staining

Paraffin-embedded sections were stained with Masson’s trichrome staining or hematoxylin–eosin staining under standard protocols. The images were collected using a light microscope (BX-51; Olympus, Tokyo, Japan). The media thickness, lumen diameter and their ratio were used as indexes of vascular remodeling.^36^

### Western blot analysis

Samples were homogenized in lysis buffer, and the supernatant was extracted for the measurement of total protein with a protein assay kit (BCA; Pierce, Santa Cruz, CA, USA). Equal amounts of total protein were separated in SDS-PAGE, and transferred to PVDF membranes in Trisglycine methanol buffer. The bands were visualized using the enhanced chemiluminescent. The primary antibodies against NLRP3, ASC, OPN, PCNA and pro-IL-1*β* were purchased from Abcam. Antibody against IL-1*β* was obtained from Proteintech (Wuhan, Hubei, China). Antibodies against H3K9ac, H3 *α*-SMA, SM22*α*, GAPDH, p65-NF*κ*B and Lamin B1 were obtained from Cell Signaling Technology (Beverly, MA, USA). Caspase-1 antibody which show caspase-1 at 10 kDa and procaspase-1 at 45 kDa as well as the antibodies against CBP and p300 were purchased from Santa Cruz Biotechnology (Santa Cruz, CA, USA).

### Real-time PCR

Total RNA was separated with a using Trizol reagent (Life Technologies) according to the manufacturer’s protocols. Reverse transcriptase reactions were performed using the PrimeScript RT reagent Kit according to the manufacturer’s instruction. Real-time PCR was performed using Quantitative PCR with SYBR Premix Ex Taq (Takara, Otsu, Shiga, Japan) and ABI PRISM 7500 sequence detection PCR system.^37^ The mRNA expression was calculated using the comparative cycle threshold (Ct) method where the relative quantization of target transcript levels was determined by subtracting Ct values of target genes from Ct values of GAPDH. The sequences of primers are listed in the supplementary tables (Supplementary Table S1).

### Intragastric administration of curcumin

WKY and SHR aged at 12 weeks were subjected to intragastric administration of polyethylene glycol (vehicle) or curcumin 100 mg/kg/day for 2 weeks as previous report.^38^

### Ad-NLRP3-shRNA transfections in VSMCs and rats

Recombinant adenovirus harboring shRNA against NLRP3 (Ad-NLRP3-shRNA) and scrambled shRNA were commercially constructed by CayGene Technology (Shanghai, China). The targeted sequence for NLRP3 and the negative control sequence were reported previously.^39^ For *in vitro* studies, VSMCs were subcultured in six-well plates and transfected with adenovirus-mediated shRNA against NLRP3 or scrambled shRNA (1 × 10^8^ PFU/ml) for 48 h. For *in vivo* studies, WKY and SHR aged at 12 weeks were subjected to receive 1 × 10^10^ plaque-forming units of an adenovirus carrying NLRP3 shRNA or scrambled shRNA via the tail vein, respectively. Final experiments were performed 4 weeks after intervention.

### VSMCs proliferation assay

VSMC proliferation was evaluated using Cell counting kit-8 kits (CCK-8; Beyotime Institute of Biotechnology, Shanghai, China) according to the manufacturer’s instructions.^40^ The absorbance was conducted at 450 nm using a microplate reader (ELX800; BioTek, Winooski, VT, USA).

### EdU incorporation assay in VSMCs

VSMC proliferation was further evaluated with EdU incorporation assay with *In Vitro* Imaging Kit (Guangzhou RiboBio, Guangzhou, China). The DNA synthesis of VSMCs was measured using a Cell-Light EdU Apollo488. The EdU-positive cells were counted and normalized by the total number of Hoechst 33342-stained cells.^40^

### EdU staining in aorta sections of rats

Intraperitoneal injection of EdU at a dose of 100 mg/kg was carried out 72 h before the thoracic aorta was harvested as previously described.^41^ The tissues were fixed in 4% formaldehyde, embedded in paraffin and transversely cut into 5-*μ*m sections using a cryostat (Leica). The EdU staining for thoracic aorta was performed using Cell-Light EdU Kit (Guangzhou RiboBio), according to the manufacturer’s protocols.^41, 42^ Paraffin-embedded sections were rinsed in 2 mg/ml glycine solution for 10 min after deparaffinization and rehydration, and the sections were then permeabilized with permeablizing with 0.5% Triton X-100 in PBS for 10 min. The 1 × Apollo reaction buffer liquid was added and incubated at 37 °C for 30 min in a dark place. The incubated sections were washed twice with PBS for 10 min each rinse. Hoechst 33342 was used to label nucleus for 30 min without light. The EdU-positive cells were observed and photographed under a fluorescent microscope (DX51; Olympus), and quantified by counting six randomly chosen high-power fields and normalized by the total number of Hoechst 33342=stained cells.

### Reporter gene transfection and luciferase activity assay

VSMCs were cultured on a 35 mm dish prior to transfection; the confluent cells were cotransfected with firefly luciferase reporter of NF*κ*B containing a TA promoter (1.0 g, pNF_BTA-luc, Beyotime Biotechnology) along with the Renilla luciferase reporter (0.1 *μ*g, Promega Co., Madison, WI, USA) for 6 h by using Lipofectamine 2000 (Invitrogen, Carlsbad, CA, USA) according to the manufacturer’s instructions. The firefly luciferase activity was measured using a dual luciferase reported gene assay kit (Beyotime Biotechnology) 24 h after transfection.^43^

### Caspase-1 activity assay

The caspase-1 activity was determined with a commercial kit according to the manufacturer’s description.^44^ In short, the standard product *p*-nitroaniline (pNA) was diluted into various concentrations to obtain a standard curve. The lytic cytosolic protein was added into acetyl-Tyr-Val-Ala-Asp *p*-nitroaniline (Ac-YVAD-pNA), and incubated for 2 h at 37 °C. The absorbance was conducted at 450 nm using a microplate reader. The production of pNA in each sample was indicated for caspase-1 activation. The results were defined as the relative value to the control.

### HAT activity assay

HAT activity was detected with a HAT assay kit (Sigma-Aldrich) as previously report.^45^ In brief, the immunocomplexes was added into HAT Assay Buffer, HAT Substrate I, HAT Substrate II and NADH Generating Enzyme, respectively. The mixtures were mixed by gently pipetting and incubated at 37 °C for 3 h. The collected supernatant from each sample was transferred to a 96-well plate and optical density was measured at 440 nm. HAT activity was expressed as the mean of the optical density, and normalized to the control.

### Enzyme-linked immunosorbent assay

IL-1*β* levels were determined using a commercial ELISA kit (Boster Biological Technology, Wuhan, China) according to the manufacturer’s protocols as previously described.^46^ Optical density was read at 450 nm using a Microplate Reader (STNERGY/H4; BioTek).

### Chromatin immunoprecipitation

ChIP was conducted as described previously.^47, 48^ Briefly, the cells or tissues were crosslinked with 1% formaldehyde for 10 min, and stopped with 125 mM glycine. Then, the samples were washed, scraped and collected. The pellets was lysed in lysis buffer (150 mM NaCl, 25 mM Tris pH 7.5, 1% Triton X-100, 0.1% SDS, 0.5% deoxycholate) supplemented with protease inhibitors. The aliquots of lysates in each chromatin solution underwent immunoprecipitation with anti-Pol II or anti-p65 antibody (Santa Cruz Biotechnology). Anti-acetyl histone H3, (Millipore, Darmstadt, Germany) or pre-immune IgG overnight at 4 °C. For re-ChIP, immunoprecipitated genomic DNA (gDNA) was eluted with the elution buffer (1% SDS, 100 mM NaCO_3_), diluted with the re-ChIP buffer (1% Triton X-100, 2 mM EDTA, 150 mM NaCl, 20 mM Tris pH 8.1). A quantitative PCR assay was implemented on the precipitated genomic DNA with primers specific for the NF*κ*B and Pol II binding site upstream of the transcriptional start site of NLRP3 and normalized against total input genomic DNA. The primer sequences (sense 5′-GCTGCAACAGTAATGATGGTGA-3′ and antisense 5′-TCAAAGCCCTAGACCAAGACT-3′) spanning the predicted consensuselements of NF-*κ*B-binding motif within the NLRP3 promoter (−594 to −293 upstream of the transcription start site) was designed with the aid of the programs TESS (available at http://www.cbil.upenn.edu/tess) and TFSEARCH (available at http://mbs.cbrc.jp/research/db/TFSEARCH.html).

### Construction of NLRP3 luciferase reporter plasmids, transfection and assay

NLRP3 promoter constructs harboring serial deletions were constructed to demarcate the region on NLRP3 promoter where NLRP3 exerts its actions in VSMCs in hypertension. The full-length promoter region of the NLRP3 gene from −2995 bp to the transcription start site, and other NLRP3 promoter fragments from −2995 to −1498, −1497 to −1, −895 to −1, −594 to −1, and −293 to −1 were amplified by PCR and were cloned into the pGL3 luciferase vector (Promega). The NLRP3 promoter luciferase vector and its deletion mutants were cotransfected with lipofectamine 2000 transfection reagent (Invitrogen). The firefly luciferase activity was measured using a dual luciferase reported gene assay kits (Beyotime) 24 h after transfection.^49^

### Statistical analysis

Comparisons between two groups were made by Student's *t-*test. ANOVA followed by *post hoc* Bonferroni test was used when multiple comparisons were made. All data were expressed as mean±S.E. A value of *P*<0.05 was considered statistically significant.
