# Supplementary material for: NLRP3 inflammasome activation contributes to VSMC phenotypic transformation and proliferation in hypertension
Source: Cell Death Dis. 2017 Oct 5;8(10):e3074–. doi: 10.1038/cddis.2017.470 (PMC5680591; doi:10.1038/cddis.2017.470)
Supplement: Supplementary Materials [file cddis2017470x1.docx]

**Supplementary Materials**

(8 supplementary figures and 1 supplementary table)

**NLRP3 inflammasome activation contributes to VSMC phenotypic
transformation and proliferation in hypertension**

Hai-Jian Sun^1,2^, Xing-Sheng Ren^1^, Xiao-Qing Xiong^1^, Yun-Zhi Chen^1^, Ming-Xia Zhao^1^, Jue-Jin Wang^1^, Ye-Bo Zhou^1^, Ying Han^1^, Qi Chen^3^, Yue-Hua Li^3^, Yu-Ming Kang^4^,
Guo-Qing Zhu^1,3^*

^1^Key Laboratory of Cardiovascular Disease and Molecular Intervention, Department of Physiology, Nanjing Medical University, Nanjing, Jiangsu 210029, China; ^2^Department of Basic Medicine, Wuxi School of Medicine, Jiangnan University, Wuxi, Jiangsu 214122, China; ^3^Department of Pathophysiology, Nanjing Medical University, Nanjing, Jiangsu 210029, China; ^4^Department of Physiology and Pathophysiology, Cardiovascular Research Center, Xi'an Jiaotong University School of Medicine, Xi'an, Shanxi 710061, China

**Supplemental Figures**

**
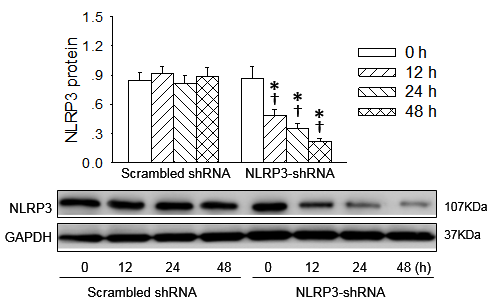
**

**Supplementary Figure 1** Time effect of NLRP3 knockdown on NLRP3 protein expression in VSMCs of SHR. Values are mean±SE. *P<0.05 vs. 0 h; †P<0.05 vs. Scrambled shRNA. n=4.

**
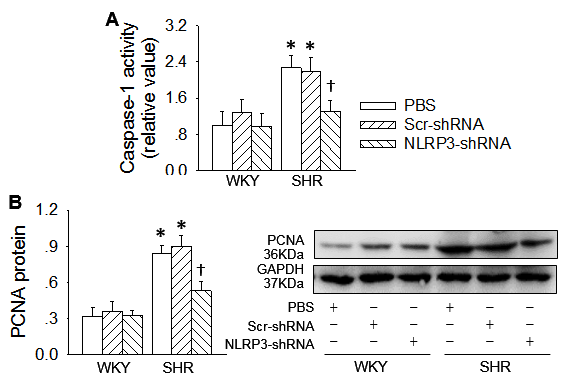
**

**Supplementary Figure 2** Effects of NLRP3 knockdown on caspase-1 activity and PCNA protein expression in VSMCs from aortas of WKY and SHR. (A) Caspase-1 activity. (B) PCNA protein expression. Values are mean±SE. *P<0.05 vs. WKY; †P<0.05 vs. PBS or Scrambled (Scr-) shRNA. n=6.

**
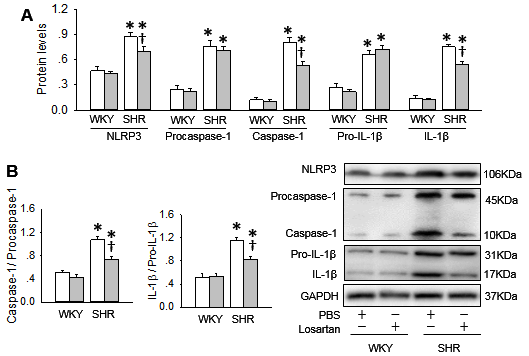
**

**Supplementary Figure 3** Effects of an AT_1_ receptor antagonist losartan (10 μM for 24 h) on NLRP3 inflammasome activation in VSMCs from aortas of WKY and SHR. (A) Relative protein expressions of NLRP3, procaspase-1, caspase-1, pro-IL-1β and IL-1β. (B) Ratio of caspase-1 to procaspase-1 and ratio of IL-1β to pro-IL-1β. Values are mean±SE. *P<0.05 vs. WKY; †P<0.05 vs. PBS. n=4.

**
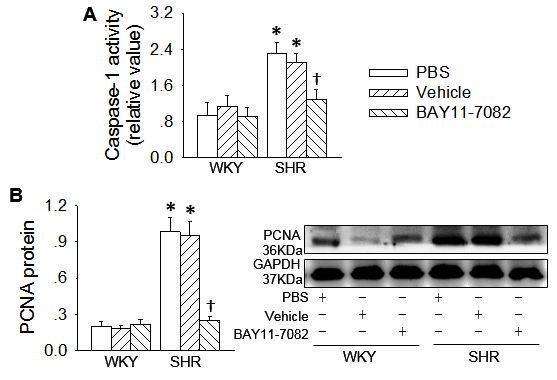
**

**Supplementary Figure 4** Effects of a NFκB inhibitor BAY11-7082 (10 μM for 48 h) on caspase-1 activity and PCNA protein expression in VSMCs from aortas of WKY and SHR. (A) Caspase-1 activity. (B) PCNA protein expression. Values are mean±SE. *P<0.05 vs. WKY; †P<0.05 vs. PBS or Scrambled (Scr-) shRNA. n=6.

**
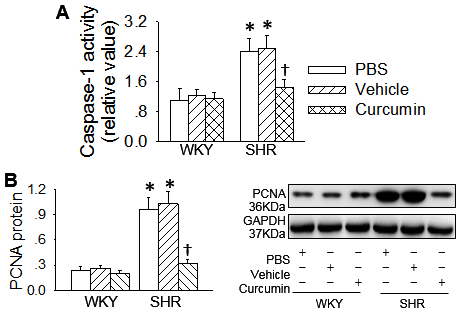
**

**Supplementary Figure 5** Effects of a histone acetyltransferase inhibition curcumin (20 μM for 48 h) on caspase-1 activity and PCNA protein expression in VSMCs from aortas of WKY and SHR. (A) Caspase-1 activity. (B) PCNA protein expression. Values are mean±SE. *P<0.05 vs. WKY; †P<0.05 vs. PBS or Scrambled (Scr-) shRNA. n=6.

**
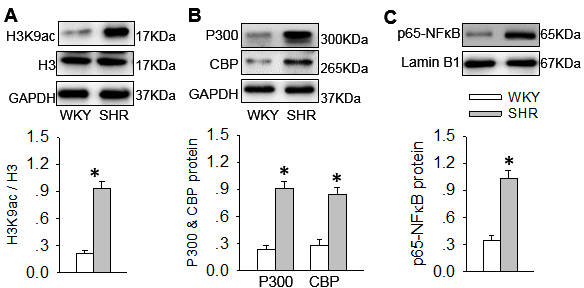
**

**Supplementary Figure 6** Histone acetylation (A), histone acetyltransferase CBP and P300 expression (B) and p65-NFκB expression in nucleus (C) in media of aorta of WKY and SHR. Values are mean±SE. *P<0.05 vs. WKY. n=4.

**
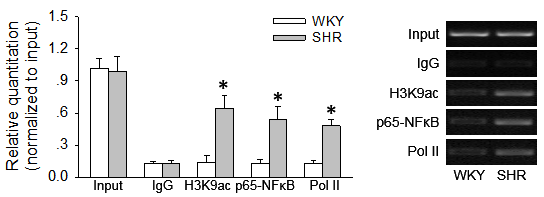
**

**Supplementary Figure 7** Enrichment of acetylated histone H3K9, p65 and Pol II in the NLRP3 promoter in media of aortas from WKY and SHR. Values are mean±SE. *P<0.05 vs. WKY. n=4.

**
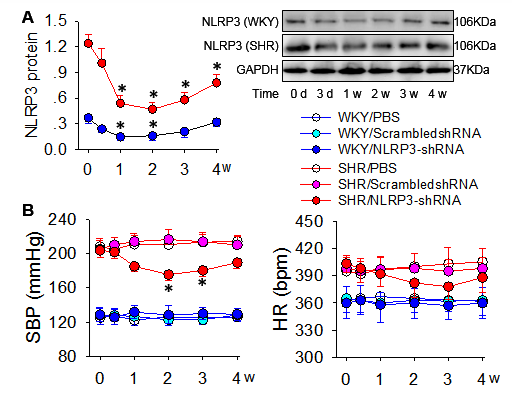
**

**Supplementary Figure 8** Effect of NLRP3 knockdown with Ad-NLRP3-shRNA in WKY and SHR. A, time effect of NLRP3 knockdown on NLRP3 expression in aortic media of WKY and SHR. Values are mean±SE. * P<0.05 vs. 0 w. n=6. B, Effects of NLRP3 knockdown on systolic blood pressure (SBP) and heart rate (HR) in WKY and SHR. Values are mean±SE. * P<0.05 vs. PBS or Scrambled shRNA. n=6.

**Supplemental Table**

**Supplementary Table 1** Primers for real-time quantitative PCR analysis in rats

|  | Primer | Sequence | Accession number |
| --- | --- | --- | --- |
| NLRP3 | Forward | 5'-GTGGAGATCCTAGGTTTCTCTG-3' | [XM_006246458.2](http://www.ncbi.nlm.nih.gov/entrez/viewer.fcgi?db=nucleotide&id=672067732) |
|  | Reverse | 5'-CAGGATCTCATTCTCTTGGATC-3' |  |
| ASC | Forward | 5'-CTCTGTATGGCAATGTGCTGAC-3' | [NM_172322.1](http://www.ncbi.nlm.nih.gov/entrez/viewer.fcgi?db=nucleotide&id=27229293) |
|  | Reverse | 5'-GAACAAGTTCTTGCAGGTCAG-3' |  |
| Caspase-1 | Forward | 5'-GAGCTGATGTTGACCTCAGAG-3' | [NM_012762.2](http://www.ncbi.nlm.nih.gov/entrez/viewer.fcgi?db=nucleotide&id=31542340) |
|  | Reverse | 5'-CTGTCAGAAGTCTTGTGCTCTG-3' |  |
| IL-1β | Forward | 5'-TGCTGTCTGACCCATGTGAG-3' | [NM_031512.2](http://www.ncbi.nlm.nih.gov/entrez/viewer.fcgi?db=nucleotide&id=158186735) |
|  | Reverse | 5'-GTCGTTGCTTGTCTCTCCTTG-3' |  |
| GAPDH | Forward  Reverse | 5'-GGAAAGCTGTGGCGTGAT-3'  5'-AAGGTGGAAGAATGGGAGTT-3' | NM-017008.4 |
